# Mobile Phones May Not Bridge the Digital Divide: A Look at Mobile Phone Literacy in an Underserved Patient Population

**DOI:** 10.7759/cureus.4104

**Published:** 2019-02-20

**Authors:** Disha Kumar, Vagish Hemmige, Michael A Kallen, Thomas P Giordano, Monisha Arya

**Affiliations:** 1 Internal Medicine, Baylor College of Medicine, Houston, USA; 2 Internal Medicine, Montefiore Medical Center, Bronx, USA; 3 Medical Social Sciences, Northwestern University Feinberg School of Medicine, Missouri City, USA

**Keywords:** text messaging, mhealth, digital divide, health literacy

## Abstract

Purpose: Mobile health (mHealth) has promise to improve patient access to disease prevention and health promotion services; however, historically underserved populations may have poor access to mobile phones or may not be aware of or comfortable using phone features. Our objectives were to assess mobile phone ownership and mobile phone literacy among low-income, predominately racial and ethnic minority patients.

Materials and methods: We conducted a cross-sectional survey of a convenience sample of primary care patients in a publicly-funded clinic in Houston, TX.

Results: Of 285 participants, 240 owned a mobile phone and 129 owned a smartphone. The most common uses of phones were talk (89%) and text messaging (65%). Only 28% of smartphone owners had health apps. Younger age was significantly associated with smartphone ownership and use of smartphones for Internet browsing, social media, and apps.

Conclusion: Our findings from a safety-net patient population represent trends in mobile phone ownership and literacy. Despite the single-site location of our study, the findings could be helpful to health promotion practitioners working with similar underserved populations. mHealth interventions should employ phone features that are accessible and familiar to the target audience to avoid denying intervention benefits to those with low mobile phone literacy and therefore widen health disparities.

## Introduction

The use of mobile devices, including mobile phones, to improve health is termed mobile health (mHealth). Although mobile phones and smartphones are widely prevalent, people with limited technology skills may be reluctant to use or unable to access technology to acquire health information on their phones [1-2]. It is critical for mHealth interventions to consider how sub-populations use mobile phones. Employing features of mobile phones that are unfamiliar may exclude populations from the benefit of these interventions and thereby worsen the “digital divide.” The term “digital divide” describes disparities in technology use [3-4]. Adapting the definition of health literacy from Selden et al., we propose the term mobile phone literacy to describe the ability to access, process, understand, and use the features on mobile phones (e.g., sending text messages, taking photos/videos, using apps) [5]. There is a knowledge gap in understanding how historically underserved and under-researched populations access and use mobile phones. The objectives of this study were to 1) assess mobile phone ownership and literacy in a low-income, predominantly racial and ethnic minority patient population, and 2) determine if there were age, income, and education discrepancies in mobile phone ownership and literacy.

## Materials and methods

Design and sample

The study took place from June 2014 to February 2015 in a primary care clinic in Houston, Texas. This primary care clinic functions within a large publicly-funded safety-net health system that serves patients who are predominately racial and ethnic minorities, low-income, and without health insurance. Participants were recruited from a convenience sample of patients in the primary care waiting room. Eligibility included being a patient at the primary care clinic and being 18 years of age or older.

Measures

Participants gave verbal consent and filled out a paper survey containing questions about mobile phone ownership, usage, reach, and preference for health information. Survey questions were adapted from national surveys administered by the Pew Research Center or developed by the research team [1]. Standard demographic questions were included. Participants could receive a $10 gift card upon survey completion. The Baylor College of Medicine Institutional Review Board approved this study under protocol H-30232.

Analysis

Two research assistants independently entered survey answers into a Microsoft ACCESS database (Microsoft Corporation, Redmond, WA) and then imported the data into Stata 13 (StataCorp, College Station, TX). Stata was used to systematically identify all discrepancies in data entry. The research team mediated discrepancies by referring to the original paper-based survey documents. Standard descriptive statistics were calculated for the analysis (i.e., means, standard deviations, student t-tests). Chi-squared analyses or Fisher’s exact test, as appropriate, were used to compare differences in outcomes of interest between demographic groups [6]. The threshold for statistical significance was set at p <0.05. No adjustment was made for multiple comparisons, as this was an exploratory study. We conducted analyses for the following defined demographic groups: younger adults (less than 50 years of age) vs. older adults (50 years of age or older); very low income individuals (household income under $20,000 annually) vs. low-to-moderate income individuals (household income $20,000 or higher annually); low education individuals (at most a high school education level) vs. high education individuals (at least some college education).

## Results

There were 285 participants who completed our survey. See Table 1 for demographics.

**Table 1:** Demographics of the Study Population (N=285*)

	n	%
Gender (n=284)		
Men	91	32
Women	193	68
Age (n=282)		
18-29	19	7
30-49	84	30
50-64	163	58
65+	16	6
Race (n=272)		
African-American	118	43
White	103	38
Asian	10	4
American Indian/Alaskan Native	1	<1
Native Hawaiian/Pacific Islander	1	<1
Other**	39	14
Ethnicity (n=285)		
Hispanic	62	22
Not Hispanic	223	78
Annual household income (n=277)		
< $20,000	207	75
$20,000-$24,999	30	11
$25,000-$29,999	18	6
$30,000-$34,999	8	3
$35,000-$39,999	7	3
$40,000-$49,999	5	2
$50,000-$59,999	1	<1
$60,000-$74,999	1	<1
Highest education level (n=281)		
No high school	10	4
Some high school	39	14
High school grad/GED	93	33
Some college	94	33
College grad	36	13
Postgraduate	9	3
*Not all participants answered all demographic questions. The “n” for each demographic category is given for the total number of participants who answered the demographic question.		
** Five participants who provided the free text response of the 39 participants who answered “Other”. One participant stated “American Mexican,” three participants stated “Hispanic,” and one participant stated “Mexican American.”		

There were 282 participants who answered the question pertaining to mobile phone ownership. Of these participants, 240 (85%) owned mobile phones. Of the 240 who owned mobile phones, 129 (54%) stated their mobile phone was a smartphone. Among the 129 respondents who answered that the type of phone they owned was a smartphone, the two most common types of smartphones were Android (n=78, 60%) and iPhone (n=22, 17%). Of the 240 mobile phone owners, 164 (68%) had an unlimited texting plan, 176 (73%) participants reported never changing their phone number in the past year, and 191 (80%) of participants had constant cell service in the past year. See Table 2 for additional details.

**Table 2:** Activities and Utilization of Mobile Phones among Mobile Phone Owners (N=240) N=number

Survey Question	Answer Choices	n	%
What do you use your mobile phone for?*	Talk	213	89
	Text messaging	155	65
	Internet browsing	83	35
	E-mail	73	30
	Photos or videos	97	40
	Apps (mobile applications)	58	24
	Social media	52	22
	Other	34	14
What kind of texting plan do you have?	Unlimited texting	164	68
	Limited texting	37	15
	Pay for each text I send and receive	8	3
	I don’t know	20	8
	Did not answer/missing	11	5
How often in 2013 did your mobile phone number change?	Never	176	73
	Once	39	16
	More than once	19	8
	Did not answer/missing	6	3
For how much of the year did you have cell service?	All of 2013	191	80
	Most of 2013	24	10
	Half of 2013	6	3
	Less than half of 2013	13	5
	Did not answer/missing	6	3
*Patients could select more than one answer			

Mobile phone activities

Of the 240 participants who owned mobile phones, 213 (89%) used their phones for talking, 155 (65%) for text messaging, and 97 (40%) for taking photos or watching videos. Study participants were less likely to use their phones for social media, Internet browsing, apps, and email, with fewer than 40% of participants engaging in these activities (Table 2). Among participants with smartphones (n=129), only 36 (28%) had health apps, which were study defined as “apps that help you take care of your health.” Of these 36 with health apps, only 8 (23%) reported using their health apps more than once a week. Notably, among the 111 mobile phone owners who did not own smartphones, 55 (50%) reported that they would be interested in having health apps on their phone.

Age, income, race/ethnicity, and education differences in smartphone ownership

Age was significantly associated with differences in smartphone ownership. While 63 of 87 (71%) mobile-phone owning younger participants owned smartphones, only 66 of 147 (45%) of older participants owned smartphones (p-value <0.01). Income was also significantly associated with smartphone ownership. Those with low income (n=43, 68%) were more likely than those with very low income (n=85, 50%) to own smartphones (p-value <0.05). Neither race/ethnicity nor education level predicted smartphone ownership.

Age, income, race/ethnicity, and education differences in mobile phone activities

Age was significantly associated with the use of smartphones for Internet browsing, social media, and apps. Younger smartphone-owning participants (n=38, 60%) were more likely than older smartphone-owning participants (n=25, 38%) to use their phone for Internet browsing (p-value <0.05). Similarly, younger smartphone-owning participants (n=28, 44%) were more likely than older smartphone-owning participants (n=15, 23%) to use their phones for social media (p-value <0.01). Finally, younger smartphone-owning participants (n=35, 56%) were more likely than older smartphone-owning participants (n=16, 24%) to use their phone for apps (p-value <0.01). Education was significantly associated with the use of text messaging. Mobile phone owners with higher education levels (n=88, 72%) were more likely than those with lower education levels (n=67, 57%) to use their phones for text messaging (p-value <0.05).

Age did not predict mobile phone use of talk or text messaging or of smartphone use of email or photos or videos. Education level did not predict mobile phone use of talk or of smartphone use of Internet browsing, social media, apps, email, or photos or videos. Neither income level nor race/ethnicity predicted mobile phone use of talk or text messaging, or of smartphone use of Internet browsing, social media, apps, email, or photos or videos.

## Discussion

While nearly all of these predominately low-income, racial and ethnic minority patients had mobile phones, many were not using their phones to full capability. Our findings highlight that text messaging or talk remains the best way to reach patients in urban safety-net outpatient settings. mHealth interventions using apps would be applicable to younger and relatively wealthier patients. Notably, our study population had slightly less mobile phone ownership compared to persons with similar demographics from the Pew Research Center national study [1]. This important finding demonstrates how national data may not reflect the hardest-to-reach populations and highlights the need for continual purposive sampling of underserved populations.

It is important to remember that mHealth interventions – such as text messaging -- may not reach all members of the target population if text messaging imposes an undue financial burden or is not routinely accessible. A 2016 study by the National Cancer Institute highlighted that age, income, and education among other socioeconomic factors play a role in inclination to use mHealth for the exchange of health information [7]. We found that a quarter to one-third of participants did not have an unlimited texting plan, changed their phone number in the past year, or lost cell service for a period of time in the past year. Further, over one in three participants in our study population did not use their phones for text messaging. These financial-driven factors demonstrate decreased access to text messaging, despite ownership of the mobile phone. A previous study found that 29% of participants would not be willing to pay for a health text message depending upon the cost of the text and the usefulness of the message [8]. Another study found that participants cited cost as a barrier to receiving text message reminders for immunizations [9].

In our low-income, racial and ethnic minority patient population ownership of mobile phones and smartphones was relatively high; however, many patients do not use their phones to full capacity. We attribute these findings to low mobile phone literacy, defined as decreased ability to access, process, understand and use mobile phone features. Those with higher education levels were more likely to use text messaging, compared to those with lower education levels. Similarly, activities of smartphone use were not ubiquitous across all demographics. Even when controlling for smartphone ownership, understanding of mobile phone features differs between younger and older adults. Among smartphone owners in our study, younger adults compared to older adults were more likely to use their phones for Internet browsing, social media, and apps. As noted by Fletcher and Jensen, barriers inhibiting older adults from using smartphone activities may include physical limitations, lack of confidence in using smartphones, and unintuitive technological design [10].

As Hswen and Viswanath noted, disparities associated with the use of mobile technologies may cause mHealth to exacerbate health disparities [11]. It is thus important to consider the mobile phone literacy of target populations. Our data are representative of trends to be aware of as mHealth interventions become more complex. Text messaging or talk are accessible modalities; however, higher technology modalities, such as apps, may risk leaving outpatients with low mobile phone literacy. To avoid the widening of health disparities in the space of mHealth, mHealth campaign designers must consider mobile phone literacy when deciding which phone features to employ for health campaigns. Campaigns should, therefore, be locally driven to meet the abilities and health needs of the target audience [12]. Furthermore, before widespread deployment, mHealth interventions should be piloted with end-users to ensure that the health interventions meet the literacy of the audience [13-14].

Our findings could be applicable to other urban primary care settings that serve low-income, racial and ethnic minority patients. Our data may not be representative of patients who lack access to primary care and face further barriers to mobile phone ownership and literacy. Given the limited mHealth research conducted to date in low-income populations, our data offers a snapshot of factors that health campaign designers should consider when targeting these historically underserved populations.

## Conclusions

Our findings highlight how mobile ownership does not predict the use of all mobile phone features. To improve the applicability of mHealth interventions aimed at patients who attend safety-net clinics and may have barriers to phone access or use, potential solutions include: subsidizing costs of mobile phone plans, routine updating of patients’ phone numbers during each patient visit, providing tutorials on how to use mobile phone features, and using multiple (e.g., text message and a phone call) preferred phone modalities to reach patients. In conclusion, to avoid widening health disparities in the field of mHealth, mHealth campaign designers should consider mobile phone access and literacy.
